# Vitamin D Levels in the United States: Temporal Trends (2011–2018) and Contemporary Associations with Sociodemographic Characteristics (2017–2018)

**DOI:** 10.3390/nu16193414

**Published:** 2024-10-09

**Authors:** Anita Subramanian, Hyacinth B. Burrowes, Jelonia T. Rumph, Jesse Wilkerson, Chandra L. Jackson, Anne Marie Z. Jukic

**Affiliations:** 1 Epidemiology Branch, National Institute of Environmental Health Sciences, Durham, NC 27709, USAjukica@niehs.nih.gov (A.M.Z.J.); 2 Department of Microbiology, Immunology and Physiology, Meharry Medical College, Nashville, TN 37208, USA; 3 Women’s Reproductive Health Research Center, Department of Obstetrics and Gynecology, Vanderbilt University Medical Center, Nashville, TN 37232, USA; 4 Social and Scientific Systems, a DLH Holdings Company, Durham, NC 27703, USA; 5 Division of Intramural Research, National Institute on Minority Health and Health Disparities, National Institutes of Health, Department of Health and Human Services, Bethesda, MD 20892, USA

**Keywords:** vitamin D, NHANES, survey, diet, race/ethnicity, epidemiology

## Abstract

**Background:** The most recent vitamin D data from the National Health and Nutrition Examination Survey (NHANES) have not been examined. We used data from NHANES to describe trends in 25-hydroxyvitamin D [25(OH)D] from 2011 to 2018 and for the most recent cycle (2017–2018) to identify groups with lower levels of 25(OH)D and factors predictive of 25(OH)D. **Methods:** The 31,628 participants were weighted to represent the entire U.S. population. For each 2-year NHANES survey cycle (2011 to 2018), we calculated the weighted median (25th and 75th percentiles) of 25(OH)D and the proportion of the population within the following categories (nmol/L): <30, 30-<50, 50-<75, 75-<125, and ≥125. For 2017–2018, we stratified by demographic and behavioral factors. Multivariate linear regression identified variables predictive of 25(OH)D. **Results:** The median 25(OH)D (nmol/L) increased slightly from 2013–2014 [66.5 (25th and 75th percentiles: 51.3, 83.0)] to 2017–2018 [68.7 (52.3, 87.8)], and the prevalence of 25(OH)D <50 nmol/L decreased slightly (23.4% vs. 21.3%). In 2017–2018, characteristics associated with lower 25(OH)D were age (12–39 years), male gender, non-Hispanic Black, higher BMI, lower income and education, winter season, not taking vitamin D supplements, or “never” using sunscreen. When stratified by age, race/ethnicity, and gender simultaneously, median 25(OH)D was lowest among non-Hispanic Black females aged 12–19 (38.5 nmol/L) or 20–39 (38.9 nmol/L). Predictors of 25(OH)D level differed by race/ethnicity, e.g., increasing BMI was associated with larger decrements in 25(OH)D among Mexican Americans. **Conclusions**: This analysis is the first to examine vitamin D levels stratified by multiple characteristics simultaneously. This strategy identified populations at higher risk for health sequelae due to low levels of vitamin D. For example, high levels of deficiency were found in non-Hispanic Black females of reproductive age.

## 1. Introduction

Vitamin D is an essential nutrient known to play a role in bone metabolism by maintaining calcium and phosphorus homeostasis [1,2]. Vitamin D can be synthesized in the skin through exposure to ultraviolet-B (UVB) radiation and absorbed through dietary intake [1,2]. Vitamin D is converted in the liver into 25-hydroxyvitamin D [25(OH)D], the major circulating metabolite and clinical biomarker for measuring vitamin D status [2,3]. Other influences on vitamin D levels include season, supplement intake, sunscreen use, skin pigmentation, and clothing practices [4,5]. Another factor affecting vitamin D levels is latitude. Although latitude can affect vitamin D levels, a study conducted in the United States reported no changes in cutaneous synthesis with varying latitudes during the summer (March–October) [6]. Although the role of vitamin D in maintaining or improving bone health is well known [1], emerging research has examined its function in non-skeletal health factors such as fertility and reproductive outcomes [7,8], osteoporosis [9], cardiovascular disease [10], diabetes [10], and immune function [11], among others. 

Vitamin D deficiency has increasingly become a global health concern [12]. The Institute of Medicine defines vitamin D deficiency as 25(OH)D levels <30 nmol/L and at risk of deficiency as between 30-<50 nmol/L [13]. In a recent meta-analysis including 308 studies published between 2000 and 2022, the global prevalence of 25(OH)D levels <30 nmol/L was reported to be 15.7% and 47.9% for levels <50 nmol/L [12].

Previous studies have described vitamin D status in the U.S. population using the National Health and Nutrition Examination Survey (NHANES) from 1988 to 2010 [14], 2007 to 2010 [15], 2011 to 2014 [16], and 2001 to 2018 [17]. Although some previous studies have examined trends in vitamin D levels across time, they have not included the most recent data cycle [14,16], or recent data were included but did not examine median 25(OH)D levels, which helps to more effectively understand 25(OH)D distributions at a population level [17]. 

Similarly, while previous studies examined differences in 25(OH)D levels across demographic characteristics, they did not examine all racial/ethnic groups (only included Hispanic, non-Hispanic White, and non-Hispanic Black), as skin pigmentation affects vitamin D levels, or they combined data from several survey years so that the most contemporary data were not described independently.

To address these literature gaps, the objectives of this study were to use NHANES data to (1) describe trends in 25(OH)D levels and vitamin D status in the U.S. population across the most recent survey years of 2011–2018 and (2) to describe contemporary 25(OH)D levels measured in 2017–2018—the most recent survey cycle. To identify groups at the highest risk of low vitamin D, the latter analysis was stratified both individually and simultaneously by demographics and behaviors, including age, gender, race and Hispanic origin (ethnicity), and BMI. Finally, we aimed to identify factors that remained independent predictors of vitamin D status after mutual adjustment. To our knowledge, this would be the first study to report 25(OH)D at the intersection of age, race/ethnicity, and gender. Findings from this study would allow us to identify specific groups with the highest risk of vitamin D deficiency and with the greatest need for intervention.

## 2. Materials and Methods

### 2.1. Study Design

**Figure 1:**
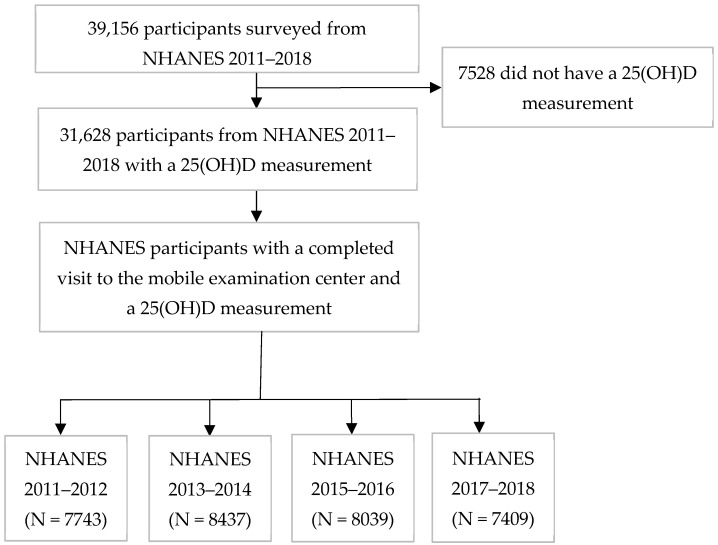
Flowchart of participants in NHANES 2011–2018. The number of participants shown represents the unweighted Ns.

NHANES is a series of biannual national surveys conducted by the National Center for Health Statistics (NCHS), a part of the Centers for Disease Control and Prevention (CDC) [18]. NHANES utilizes a complex survey design and a multistage probability sampling method to collect data representative of the non-institutionalized civilian U.S. population [19,20]. NHANES includes questionnaires, examinations, and laboratory results for adults and children in the United States [18,19,20,21,22,23]. Oversampling is used to ensure representation of certain groups such as African Americans, Hispanics, Asians, and older adults (>60 years) [24]. NHANES participants were interviewed in their homes and provided demographic information, including age, gender, race/ethnicity, annual household income, and educational attainment. Gender was reported by a household member and categorized as “male”, “female”, “don’t know”, or “refused”. Race/ethnicity was based on self-identification and categorized into the groups of “non-Hispanic White”, “non-Hispanic Black”, “non-Hispanic Asian”, “Mexican American”, “Hispanic non-Mexican”, and “Another Race” (represents the “Other” race category defined by NHANES, which includes individuals belonging to multiple races or other races such as Central American, South American, Puerto Rican, Cuban, or Dominican Republic) [19,20,25]. Data were collected on other factors, including dietary vitamin D, supplement intake, and sunscreen use. Participants took part in a detailed dietary interview estimating the food and beverages they consumed in the last 30 days, which was processed by NCHS coders to estimate vitamin D from all sources (vitamin D2 + D3) in micrograms (mcg) [26,27]. Dietary data collected were coded to represent different food groups (milk and milk products, fruits, vegetables, etc.) by combining similar foods and beverages together. In addition, combination codes were used for grouping foods that were eaten together, such as cereal with milk or ice cream with toppings [28]. Supplement data included the participants’ reported 30-day use of prescription and non-prescription dietary supplements, including their total vitamin D (mcg) intake. Sunscreen use was reported based on use during a sunny day when participants spent more than an hour outside and was categorized as “always”, “most of the time”, “sometimes”, “rarely”, “never”, “refused”, or “don’t know”. Smoking status was categorized into three groups: “current”, “former”, and “never”. Participants reported the frequency of alcohol intake during the past 12 months, and this variable was categorized into two groups: “yes” and “no”. They also reported whether they were diagnosed with diabetes or kidney disease. Participants provided blood samples during the survey visit to the mobile examination center. This analysis includes NHANES 2011–2018 data, including survey years 2011–2012 (7743 participants), 2013–2014 (8437 participants), 2015–2016 (8039 participants), and 2017–2018 (7409 participants), where 25(OH)D from blood samples were available (Figure 1). A total of 31,628 participants had 25(OH)D concentrations measured in the 2011–2018 surveys. 

### 2.2. Laboratory Methods

For NHANES survey cycles 2011–2018, serum vitamin D metabolites were quantified using high-performance liquid chromatography–tandem mass spectrometry (HPLC-MS/MS) [29]. This method can quantify 25(OH)D_2_, 25(OH)D_3_, and 3-epi-25(OH)D_3_ (epi-2(OH)D_3_). Total serum 25(OH)D [25(OH)D_2_ + 25(OH)D_3_] was used for our analysis. Quality control was tested with blind controls, which included serum samples reflecting both the high and low levels of 25(OH)D, or bench controls, which included serum samples representing low, medium, and high 25(OH)D) levels. Quality control was further tested using a defined set of criteria described elsewhere [29]. Coefficient of variation for quality control samples measured in NHANES 2017–2018 for 25(OH)D_2_ was ≤4.4%, and that for 25(OH)D_3_ was ≤2.5%. Participants living in the northern latitudes participated in NHANES in the summer (May–October), while participants living in the southern latitudes were recruited in winter (November–April). We included season of blood collection in our analysis, although we acknowledge that season is correlated with latitude.

### 2.3. 25(OH)D Categories

The Institute of Medicine categories for vitamin D using serum 25(OH)D were used for our analyses to define deficiency and at risk of deficiency [13]. The Institute of Medicine definition of deficiency is <30 nmol/L (<12 ng/mL), and “at risk of deficiency” is 30–49 nmol/L (12–19 ng/mL) [13]. We included another category to represent 25(OH)D levels between 50–75 nmol/L (20–30 ng/mL). Two additional categories were included to represent higher levels of 25(OH)D, 75–125 nmol/L (30–40 ng/mL), and ≥125 nmol/L (≥50 ng/mL), which have been associated with health benefits [8,30,31], for a total of five categories.

### 2.4. Statistical Analysis

The data was weighted to represent the U.S. population. The weighting accounts for missing data [19,20]. First, we calculated weighted median, 25th, and 75th percentiles of 25(OH)D levels and weighted prevalences of 25(OH)D categories across survey years 2011–2018. Since vitamin D may have a skewed distribution, we described the results using median (25th and 75th percentile) to better describe central tendency. Weights were applied to represent the entire U.S. population. Second, for NHANES 2017–2018, the weighted prevalence of 25(OH)D categories and weighted median (25th and 75th percentiles) 25(OH)D were reported stratified by demographic and behavioral variables and across several cross-stratifications. The goal of these cross-stratifications was to investigate whether patterns seen in the univariate analyses persisted when accounting for a second or third variable. For example, we examined age, gender, and BMI stratified by race/ethnicity (bivariate analysis) to investigate whether univariate patterns in these variables persisted across racial/ethnic groups. Additional bivariate analyses were (1) age and gender by BMI, (2) gender by age, and (3) dietary vitamin D intake and supplement use by age. Our multivariate analyses included a cross-stratification by three variables: age, race/ethnicity, and gender, and a weighted multivariate linear regression model was used to identify predictors of 25(OH)D while accounting for all other potential predictors. The variables included in this regression model were age, gender, BMI, educational attainment, vitamin D supplement use, dietary intake, season, smoking, alcohol intake, diabetes, and kidney disease. Two regression models were fit: first, a linear regression model with 25(OH)D as the dependent variable and all of the previously listed variables as the independent variables, and second, the same linear regression model but with interactions included between the independent variables and race/ethnicity to examine differences in predictors across racial and ethnic groups. The observed sample size for the regression model was smaller than other analyses because it was limited to participants who had available data for both vitamin D supplements and dietary intake (N = 6165). Statistical analyses were performed using RStudio (version 2023.06.0+421), along with the *survey*, *nhanesA*, *psych*, and the *dplyr* packages to account for the complex survey design of the NHANES. Sampling weights were used to account for oversampling and nonresponse. The medical examination weights provided in the NHANES data were used for all analyses except for the calculations of 25(OH)D levels by vitamin D intake (via diet and supplements), for which the dietary weights were applied.

## 3. Results

### 3.1. Temporal Trends in 25(OH)D in 2011–2018 NHANES

In NHANES 2011–2018, there were 31,628 participants with a 25(OH)D measurement (Figure 1). In 2011–2012 (N = 7743), the median 25(OH)D was 67.9 nmol/L (25th and 75th percentiles: 51.1, 85.0) (Table 1). The subsequent years were similar in 25(OH)D levels, but in 2017–2018 (N = 7409), the median 25(OH)D was slightly higher, at 68.7 nmol/L (25th and 75th percentiles: 52.3, 87.8). Consistent with the slight increase in median 25(OH)D, the percentage of people with 25(OH)D concentrations of <50 nmol/L or less declined across the study years from 23.4 to 21.3%. At the same time, the proportion of the population with 25(OH)D greater than 75 nmol/L slightly increased across the survey years, from 39.0% in 2011–2012 to 40.1% in 2017–2018.

### 3.2. Factors Associated with 25(OH)D Levels in 2017–2018: Univariate and Bivariate Analyses

#### 3.2.1. Race/Ethnicity 

The non-Hispanic Black population had a higher prevalence of 25(OH)D levels less than 30 nmol/L compared with the next highest groups: Mexican American (21% vs. 8%) and non-Hispanic Asian (21% vs. 8%) (Table 2). All other racial/ethnic groups had 5% or less of their population in this category. Further, the non-Hispanic Black population had the lowest median 25(OH)D level, 47.7 nmol/L (25th and 75th percentiles: 32.4, 67.0). The non-Hispanic White population had the highest prevalence of 25(OH)D levels 75-<125 or ≥125 nmol/L, which were 45% and 7%, respectively, while all other race groups had less than 30% and less than 5% in these categories. 

#### 3.2.2. Age

There was a U-shaped pattern to 25(OH)D levels across age categories, with levels of 76.1 nmol/L for ages 0–4, a nadir of 59.8 at ages 20–39, and 87.0 for ages over 60 (Table 2). People in the youngest (0–4 years old) and oldest (≥60 years old) age groups were more likely to have 25(OH)D levels greater than 75 nmol/L (54% and 63%, respectively, compared with 22–43% in the other age groups). The lowest and highest ages had the lowest prevalence of levels between 30–50. The oldest age group had a high prevalence of levels ≥125 nmol/L, more than twice that of other age groups. 

When stratified by race/ethnicity, the U-shaped association between age and 25(OH)D levels was present across all racial and ethnic groups (Table 3). The group with the lowest median 25(OH)D concentration was non-Hispanic Black, aged 20–39 years [median 25th and 75th: 39.7 nmol/L (27.7, 54.7)]. 

Table 4 shows 25(OH)D levels stratified by age and BMI among the three age categories where BMI could be calculated. Differences in 25(OH)D levels across age categories (rows of Table 4) were larger than differences across BMI categories (the columns of Table 4). Among individuals aged 20–39 and 40–59, the lowest median 25(OH)D level was in the highest BMI category (≥30 kg/m^2^) [57.2 nmol/L (25th and 75th: 42.8, 71.3) and 66.7 (25th and 75th: 50.5, 82.9), respectively]; however, there was no dose–response relationship across BMI categories. For individuals aged ≥60, a dose response relationship was not observed across BMI categories, and the lowest 25(OH)D levels were found in the lowest BMI category (<18.5) [59.0 nmol/L (25th and 75th: 40.3, 80.5)].

When 25(OH)D levels were stratified by dietary vitamin D and age, there was a dose–response relationship observed in median 25(OH)D levels with increasing intake of dietary vitamin D for all ages except the oldest group (≥60) (Table S1). However, the highest levels of 25(OH)D across all age groups were found among those who were at least 60 years or older and with a dietary vitamin D intake between 16 and 25 mcg [94.7 nmol/L (25th and 75th: 80.4, 107). Similar to dietary intake, a dose–response relationship was observed for vitamin D supplement intake among all age groups. The highest median 25(OH)D levels were found among the older population (≥60) with a vitamin D supplemental intake higher than 1000 IU [107 nmol/L (25th and 75th: 92.2, 126)].

#### 3.2.3. Gender

Overall, in the U.S. population, females had higher median 25(OH)D concentrations than males (Table 2). Females were slightly more likely to be in the two highest categories of 25(OH)D, 75-<125 and ≥125 nmol/L, with 37% and 7%, respectively, compared with 32% and 4% in males. The difference between 25(OH)D levels in males and females was small across all racial and ethnic groups, with females having slightly higher levels in all racial/ethnic groups except one (Table 3). The biggest difference between males and females was in the non-Hispanic White group (median 25(OH)D: 79.2 vs. 73.1 nmol/L).

When 25(OH)D levels were stratified by gender and BMI, females had higher levels than males across all BMI categories except the lowest BMI category, <18.5 kg/m^2^, but the differences were quite small. Among men, there was no dose–response association between median 25(OH)D levels and increasing BMI, but the lowest 25(OH)D level was found in the highest BMI category, 64.0 nmol/L (25th and 75th: 49.1, 82.8) (Table 4). Among females, the median 25(OH)D level increased across the three lower BMI categories (<18.5, 18.5–24.9, and 25–29.9) from 69.6 nmol/L (25th and 75th: 55.1, 84.1) to 76.7 nmol/L (25th and 75th: 55.6, 101.0), before dropping to 65.6 nmol/L (25th and 75th: 47.3, 89.6) for those with a BMI ≥30.

When stratified by age and gender, the U-shaped association of 25(OH)D levels with age was present for both males and females (Table 5). The nadir for males was in the 20–39 age group [median: 58.4 nmol/L (25th and 75th: 45.5, 71.2)], and the nadir for females was also in the 20–39 age group [median: 61.7 nmol/L (25th and 75th: 46.7, 78.1)]. In the oldest population (≥60), females had higher levels than males; the median 25(OH)D was 92.2 nmol/L (25th and 75th: 69.1, 110.0) vs. 79.0 nmol/L (25th and 75th: 60.4, 102.0), respectively.

#### 3.2.4. BMI

Overall, the proportion of the U.S. population with 25(OH)D <30 nmol/L or 30-<50 nmol/L increased across BMI categories (Table 2). Those with a higher BMI (≥30) had the lowest median 25(OH)D of 64.3 nmol/L (25th and 75th: 48.1, 85.8). A higher BMI (≥30) was associated with the lowest levels of 25(OH)D across all racial and ethnic groups (Table 3). Uniquely, among Mexican Americans, there was a dose–response association, with increasing BMI being associated with decreasing 25(OH)D.

#### 3.2.5. Education

Individuals who attained a high school degree or less had a lower median 25(OH)D [60.0 nmol/L (25th and 75th percentiles: 45.1, 77.6)] and a higher prevalence of low 25(OH)D (<50 nmol/L) (Table 2). Those who attained a college-level education or higher had a higher median 25(OH)D [74.4 nmol/L (25th and 75th percentiles: 58.7, 93.9)] and a lower prevalence of low 25(OH)D (<50 nmol/L).

#### 3.2.6. Other Factors

Characteristics associated with being in the highest categories of 25(OH)D were high income, supplement use, 25(OH)D measured in summer, sunscreen use “most of the time” or “always”, former smoker, and no alcohol intake (Table 2). Those without a diagnosis of diabetes or kidney disease had lower median 25(OH)D levels.

### 3.3. Factors Associated with 25(OH)D Levels in 2017–2018: Multivariate Analyses

When stratified by age, gender, and race/ethnicity simultaneously, non-Hispanic Black individuals had the lowest 25(OH)D levels across all age groups and genders (Table 6 and Figure S1). The lowest median 25(OH)D was observed among non-Hispanic Black females aged 12–19 years [38.5 nmol/L (25th and 75th percentiles: 26.9, 48.7)] and 20–39 years [38.9 nmol/L (25th and 75th percentiles: 27.1, 54.7)]. Similarly, lower 25(OH)D levels were observed among non-Hispanic Black males aged 20–39 years [40.2 nmol/L (25th and 75th percentiles: 28.3, 55.1)]. While the univariate and bivariate data suggested that females have higher levels than males, this was not true for many groups. For example, levels were also low for Mexican American females aged 12–19 years [49.6 nmol/L (25th and 75th percentiles: 37.8, 59.8) nmol/L] or aged 20–39 years [49.3 nmol/L (25th and 75th percentiles: 39.3, 64.3) nmol/L], and these were lower than their male counterparts. Similarly, in the non-Hispanic Asian group, females had lower levels than males in the younger age groups. Across racial ethnic groups, females had higher levels of 25(OH)D in the highest age groups. For example, the highest 25(OH)D levels were observed among non-Hispanic White females aged ≥60 years compared to males [median: females, 93.9 nmol/L (25th and 75th percentiles: 73.0, 112.0); males, 85.7 nmol/L (25th and 75th percentiles: 63.5, 103.0)].

### 3.4. Predictors of 25(OH)D 

In the multivariate model with mutual adjustment, the U-shaped association between age and 25(OH)D levels was maintained (Table 7). The dose–response association across BMI was clear, with a BMI of <18.5 having the highest 25(OH)D levels and ≥30 having the lowest. Vitamin D supplement use, dietary intake, survey season, current smoking, and the presence of kidney disease were also predictive of 25(OH)D.

When stratified by race/ethnicity, interaction terms between race/ethnicity and gender and race/ethnicity and education were not significant. Significant interactions were observed between race/ethnicity and age, BMI, vitamin D supplement use, dietary intake, and season. The U-shaped association between age and 25(OH)D level was accentuated among those who reported their race/ethnicity as non-Hispanic Black, non-Hispanic Asian, or Hispanic non-Mexican.

An increasing BMI was associated with larger decrements in 25(OH)D among Mexican Americans and those who reported “Another Race”, where a BMI of ≥30 was associated with an 11 nmol/L decrease in 25(OH)D compared with 5.2–5.9 nmol/L in the Hispanic non-Mexican, the non-Hispanic White, and the non-Hispanic Black groups. Among those who reported their race as non-Hispanic White, the association of supplementation with 25(OH)D levels was slightly weaker than for other race/ethnicity groups.

Among those who reported their race/ethnicity as Mexican American or Another Race, the dietary intake of vitamin D was associated with a dose–response increase in 25(OH)D. This association was not present for other racial/ethnic groups. The effect of season was strongest among those who reported their race as non-Hispanic White and was reduced by half or more among other racial/ethnic groups. Current smokers had the lowest 25(OH)D levels, and this was consistent across all racial/ethnic groups, but the decrease in levels was higher among Hispanics and non-Mexicans, followed by the non-Hispanic Black population. Alcohol intake was not associated with 25(OH)D, but a larger decrease in levels was observed among non-Hispanic Black and non-Hispanic Asian populations. There was not a clear association between diabetes and 25(OH)D, whereas those with kidney disease had higher 25(OH)D levels.

## 4. Discussion

In this nationally representative study, we summarized NHANES data across time, demographics, and health behaviors. In NHANES data from 2011 to 2018, levels of 25(OH)D increased slightly while remaining above the level of deficiency from 2013–2014 to 2017–2018. Consistent with the median increase, the proportion of people with 25(OH)D levels <50 nmol/L declined slightly across the study years, and the proportion with levels ≥125 nmol/L increased slightly. In the most contemporary data available, 2017–2018, 25(OH)D levels were associated with several demographic and behavioral characteristics both overall and within certain demographic groups. Our univariate analyses present an overall marginal summary of the associations of 25(OH)D with demographics and behaviors, which is useful for understanding the pattern across the entire U.S. population. Our multivariate and stratified analyses provide deeper insight into these associations, addressing confounding for some variables and identifying groups that appear at the highest risk for vitamin D deficiency. 

In univariate analyses, the association between 25(OH)D and age was U-shaped, with the highest levels seen in the youngest (0–4 years) and oldest (≥60 years) age groups and the lowest levels in the 12–19 and 20–39 age groups. In multivariate and stratified analyses, the U-shaped association persisted and was amplified among those who self-identified as non-Hispanic Black, non-Hispanic Asian, or Hispanic non-Mexican.

In univariate analyses, 53% of the non-Hispanic Black population had 25(OH)D concentrations of 50 nmol/L or less, and the prevalence was higher than that observed in the non-Hispanic White population (11%). Thus, half of all U.S. non-Hispanic Black people are below the Institute of Medicine cutoff for at risk of vitamin D deficiency. The Mexican American population also had lower levels of 25(OH)D compared with other race groups, with almost 40% being vitamin D deficient. In contrast, the non-Hispanic White population had the highest prevalence of 25(OH)D concentrations between 75–125 nmol/L. These findings support established racial/ethnic disparities in vitamin D levels [14,15,16]. The prevalence of 25(OH)D levels <40 nmol/L was highest among non-Hispanic Blacks for all NHANES survey cycles from 1998 to 2010 and was between 46 and 60% [14]. In NHANES 2007–2010, lower mean 25(OH)D levels were reported in the non-Hispanic Black population (46.6 nmol/L) compared to the non-Hispanic White population (75.2 nmol/L) [15]. In NHANES 2011–2014, the prevalence of at risk of vitamin D deficiency (<30 nmol/L) and at risk of inadequacy (30–49 nmol/L) among non-Hispanic Black individuals was 17.5% and 35.8%, respectively [16].

In univariate analyses, U.S. females had higher 25(OH)D levels than males and were more likely to be in the ≥75 nmol/L category; however, multivariate analyses revealed that this was driven mostly by females 60 years of age or older. At younger ages, females tended to have lower levels than males, especially in the non-Hispanic Black and Hispanic non-Mexican groups.

When stratified by age, race/ethnicity, and gender, simultaneously, 25(OH)D levels were lowest among non-Hispanic Black females aged 12–39. Within this group, the 75th percentile was around 50 nmol/L, indicating that about 75% of the population is deficient. Vitamin D may improve fertility and reproductive outcomes [32,33]. Vitamin D receptors are expressed in the ovary and placenta and may play a part in improving implantation [33], folliculogenesis [33], and placental development [34]. Studies have shown that lower levels of 25(OH)D can result in decreased fecundability [8,35], longer menstrual cycle length [30,36], delayed ovulation [30], and increased risk of miscarriage [37]. Given the potential importance of vitamin D for reproductive and pregnancy health, our findings support the need for further research targeted on reproductive-age females, particularly non-Hispanic Black females.

In univariate analyses, individuals with a BMI greater than 30 had the lowest 25(OH)D concentration levels. In multivariate analyses, we found that while increasing BMI was associated with lower 25(OH)D overall, there were larger decrements in 25(OH)D among Mexican Americans and individuals of Another Race. Our findings are similar to other studies reporting lower 25(OH)D levels among those with a higher BMI [15,17,38,39]. Although prior research exists on the storage of vitamin D in the adipose tissue among individuals with higher BMI [40,41], the findings are mixed and inconsistent and warrant further research into investigating this mechanism.

In univariate analyses, seasonal variation was observed, with a higher proportion of the U.S. population having 25(OH)D levels of 50 nmol/L or less during the winter months when sunlight exposure is decreased. In multivariate analyses, the season was a predictor of 25(OH)D, and it was at least twice as strong among those who reported their race as non-Hispanic White compared with other racial/ethnic groups. These findings are in line with what we would expect among people with lighter skin pigmentation. Seasonal variation in vitamin D levels has been previously observed [42,43], and it is hypothesized that skin pigmentation plays an essential role in the cutaneous synthesis of vitamin D [44,45]. Individuals with darker skin pigmentation have higher concentrations of melanin, which blocks UVB absorption needed for vitamin D synthesis, thereby lowering cutaneous production of vitamin D [44,45]. When exposed to UVB, lower levels of 25(OH)D were observed among individuals reporting their race as Black compared to Asian or White [44,46]. Our findings emphasize the importance of vitamin D supplementation, particularly among non-Hispanic Black, Mexican American, Hispanic non-Mexican, and non-Hispanic Asian populations. Moreover, people living at higher latitudes have lower levels of 25(OH)D due to changes in the zenith angle of the sun, which results in very little UVB reaching the Earth’s surface [4]. Thus, people belonging to high-risk racial/ethnic groups living at higher latitudes may be especially vulnerable to low levels of 25(OH)D.

Univariate analyses showed that, in general, supplementation was associated with higher 25(OH)D levels; however, multivariate analyses showed that it was slightly weaker in the non-Hispanic White population. Similarly, multivariate analyses found that dietary intake of vitamin D was associated with a dose–response increase in 25(OH)D among those who reported their race/ethnicity as Mexican American or Another Race, but this association was not present for other racial/ethnic groups.

Our study has limitations. This study is cross-sectional in design and uses some self-reported data that could be affected by recall error. Vitamin D status may change over time, and a single measurement does not allow us to assess within-person longitudinal change. Data on the season was collected in the summer among those living in the northern latitudes and in the winter among those in the southern latitudes. Our results may conflate seasonal patterns with latitude. Moreover, no data were collected in the south in the summer or in the north in the winter, which would reduce the variability observed. Data on dietary vitamin D and supplement use were only available for a subset of participants, but NHANES reweighted this subset to be nationally representative.

A strength of this study includes the large sample size representative of the U.S. population. Also, we included the most recent NHANES cycle with vitamin D measures (2017–2018) to provide the most contemporary estimates of 25(OH)D levels in the U.S. Vitamin D was measured consistently using LC-MS/MS for all the survey years included in this study. The LC-MS/MS is a robust measurement method that is highly accurate [47]. We looked at associations between vitamin D and demographic factors across several cross-stratifications using the latest available vitamin D data to determine factors that increase the risk of low vitamin D levels. We examined the findings across all racial/ethnic groups and did not limit the analysis to only a few groups. In addition to univariate analyses, we performed multivariate and stratified analyses, which provide insights into the at-risk groups and can be used to guide the design and sample size calculations of future studies targeted toward these particular groups.

## 5. Conclusions

These findings illuminate trends in 25(OH)D concentrations in the U.S. population. These data can be used to estimate sample size and power for research studies that plan to include vitamin D-deficient participants recruited from population sub-groups, such as reproductive-age females, within the U.S. While median vitamin D levels in the U.S. increased slightly from 2011 to 2018, there were disparities in the most recent years, with low levels of 25(OH)D in certain groups. Vitamin D was lowest among non-Hispanic Black females of reproductive age, suggesting that this population may be more at risk for vitamin D deficiency-related health outcomes. These results highlight the importance of examining vitamin D levels for both the U.S. population as a whole and stratified by demographic factors such as age, race/ethnicity, and BMI, and suggest the need for further research to address disparities in vitamin D status among different population groups.

## Figures and Tables

**Table 1:** Temporal trends in 25(OH)D levels in the United States: NHANES 2011–2018 ^1^.

	Weighted Median (25th and 75th Percentiles)							
	2011–2012		2013–2014		2015–2016		2017–2018	
**25(OH)D (nmol/L)**	67.9 (51.1, 85.0)		66.5 (51.3, 83.0)		67.8 (51.8, 85.4)		68.7 (52.3, 87.8)	
	**Weighted Prevalence (%)**							
	**N** ^2^	**%**	**N** ^2^	**%**	**N** ^2^	**%**	**N** ^2^	**%**
<30	553	4.9	573	5.1	488	4.4	550	4.7
30-<50	1902	18.5	1806	18.1	1977	18.1	1540	16.6
50-<75	3067	37.6	3558	40.7	3339	39.1	2886	38.5
75-<125	2030	34.7	2297	32.6	2032	33.8	2149	35.1
≥125	191	4.3	203	3.5	203	4.6	284	5.0

^1^ Mobile examination center (MEC) subgroup weights used for analysis. ^2^ Represents observed participants and not a weighted N. 25(OH)D, 25-hydroxyvitamin D.

**Table 2:** Weighted prevalence and weighted median (25th and 75th percentiles) 25(OH)D levels for the U.S. population, stratified by demographics, vitamin D supplement use, and sunscreen use, NHANES, 2017–2018 ^1^.

		25(OH)D (nmol/L) ^2^					
	N ^3^	<30 (%)	30-<50 (%)	50-<75 (%)	75-<125 (%)	≥125 (%)	Weighted Median (25th and 75th Percentiles)
Race/Ethnicity							
Mexican American	1118	8	29	48	14	1	55.5 (43.1, 68.3)
Hispanic non-Mexican	675	4	22	49	23	2	62.8 (49.2, 74.6)
Non-Hispanic White	2503	1	10	36	45	7	76.1 (61.1, 94.3)
Non-Hispanic Black	1678	21	32	29	15	2	47.7 (32.4, 67.0)
Non-Hispanic Asian	935	8	24	40	24	4	60.0 (45.8, 78.6)
Another Race	500	5	15	48	28	3	65.7 (51.9, 79.1)
Age (years)							
0–4	471	2	7	37	52	2	76.1 (64.0, 88.0)
5–11	940	1	13	54	32	0	67.5 (56.7, 79.4)
12–19	1015	7	23	49	20	2	60.9 (46.8, 73.2)
20–39	1490	7	24	43	23	2	59.8 (45.7, 75.1)
40–59	1576	5	15	37	38	5	70.7 (54.3, 88.2)
≥60	1917	2	10	25	50	13	87.0 (63.8, 107.0)
Gender							
Male	3610	4	17	42	32	4	67.0 (51.5, 83.1)
Female	3799	5	16	35	37	7	70.7 (53.0, 92.0)
BMI (kg/m^2^) ^4^							
<18.5	1079	2	13	46	38	1	70.3 (57.7, 83.6)
18.5–24.9	1988	4	15	41	34	6	69.4 (54.6, 89.0)
25.0–29.9	1832	5	16	36	38	6	71.1 (54.5, 89.8)
≥30.0	2283	7	20	37	31	5	64.3 (48.1, 85.8)
Annual household income (USD)							
0–14,999	758	8	24	38	28	3	62.8 (44.9, 82.0)
15,000–34,999	1646	5	21	38	33	4	65.9 (49.3, 85.5)
35,000–64,999	1671	6	17	38	34	4	67.4 (50.7, 84.9)
65,000 and over	2345	3	17	40	34	7	68.4 (54.1, 88.7)
Educational attainment ^5^							
<High school	1352	8	25	40	26	2	60.0 (45.1, 77.6)
High school grad/ GED or some college/ AA degree	4045	5	17	39	34	5	67.8 (51.7, 86.3)
≥College graduate	1665	3	12	36	42	8	74.4 (58.7, 93.9)
Season							
Winter months ^6^	3551	7	22	39	28	4	63.5 (47.6, 81.2)
Summer months ^7^	3858	3	12	38	40	6	72.9 (57.8, 91.8)
Supplement Use ^8^							
No	4361	7	23	44	25	1	61.4 (46.6, 75.8)
Yes	2252	1	7	29	51	11	83.6 (67.0, 104.0)
Sunscreen Use							
Never	1216	13	25	38	23	2	57.8 (39.2, 74.6)
Rarely	564	4	18	48	29	2	66.7 (52.4, 79.6)
Sometimes	634	4	20	40	32	3	66.5 (50.3, 84.5)
Most of the time	356	2	14	36	40	8	74.3 (56.6, 91.0)
Always	284	3	16	40	36	6	68.7 (54.9, 88.6)
Smoking Status ^9^							
Current	910	7	21	42	27	3	62.6 (46.9, 78.4)
Former	1212	3	13	37	40	8	73.5 (57.8, 93.3)
Never	3103	5	17	34	37	7	69.7 (51.6, 91.9)
Alcohol Use ^9^							
Yes	3340	5	16	36	37	6	69.9 (52.4, 89.7)
No	1012	3	17	33	37	9	72.7 (54.0, 95.2)
Diabetes							
Yes	814	4	15	30	43	7	75.1 (54.8, 95.3)
No	6591	5	17	39	34	5	68.2 (52.0, 87.0)
Kidney Disease ^10^							
Yes	207	4	10	20	53	14	89.3 (63.4, 108.3)
No	4768	5	17	36	36	6	69.4 (51.8, 89.7)

^1^ Mobile examination center (MEC) subgroup weights used for analysis. ^2^ Percentages are weighted to represent the U.S. population. ^3^ Number of observed participants, unweighted. ^4^ Measured for male and female participants aged ≥2 years. ^5^ Education level of the household reference person. ^6^ 1 November through 30 April. ^7^ 1 May through 31 October. ^8^ Diet and supplement subgroup weights used. ^9^ Measured for male and female participants aged ≥18 years. ^10^ Measured for male and female participants aged ≥20 years. BMI, body mass index; 25(OH)D, 25-hydroxyvitamin D.

**Table 3:** Weighted median (25th and 75th percentiles) 25(OH)D for the U.S. population, stratified by race/ethnicity: NHANES 2017–2018 ^1^.

25(OH)D (nmol/L)						
Weighted Median (25th and 75th Percentiles)						
Race/Ethnicity						
	Mexican American	Hispanic non-Mexican	Non-Hispanic White	Non-Hispanic Black	Non-Hispanic Asian	Another Race
Age (years)						
0–4	63.6 (55.9, 78.3)	73.7 (64.8, 79.5)	81.2 (71.5, 91.6)	61.3 (48.2, 76.1)	70.1 (56.3, 82.3)	74.3 (63.3, 103.0)
5–11	61.3 (51.9, 70.1)	65.1 (55.7, 71.2)	75.7 (67.1, 88.1)	54.1 (42.8, 76.1)	58.7 (52.5, 67.1)	67.6 (57.7, 79.7)
12–19	52.0 (39.6, 61.4)	55.9 (44.3, 61.7)	69.6 (59.9, 83.1)	43.6 (30.7, 51.7)	49.3 (38.8, 60.3)	61.9 (47.9, 70.2)
20–39	49.8 (38.6, 61.6)	56.8 (46.6, 69.4)	68.2 (55.3, 82.2)	39.7 (27.7, 54.7)	50.8 (38.5, 61.4)	60.7 (42.6, 77.7)
40–59	56.0 (44.1, 68.5)	68.3 (50.8, 78.8)	76.4 (62.0, 92.7)	48.2 (32.1, 70.6)	66.8 (51.2, 85.3)	69.4 (53.9, 79.6)
≥60	71.3 (54.2, 88.6)	73.3 (57.1, 90.7)	91.9 (68.4, 109.0)	70.4 (43.5, 98.5)	84.5 (67.2, 104.0)	73.5 (60.4, 106.0)
Gender						
Male	56.1 (44.2, 67.5)	61.8 (47.2, 73.7)	73.1 (60.4, 89.6)	47.4 (33.9, 63.8)	57.5 (43.4, 74.6)	65.3 (51.5, 76.3)
Female	54.8 (42.7, 69.0)	63.8 (52.3, 75.0)	79.2 (62.1, 99.0)	48.4 (31.5, 70.8)	62.0 (47.6, 83.6)	66.0 (54.3, 81.4)
BMI (kg/m^2^) ^2^						
<18.5	62.8 (53.5, 71.1)	64.9 (53.6, 73.8)	76.0 (65.2, 89.9)	52.9 (40.3, 68.6)	58.7 (48.7, 69.0)	73.6 (61.3, 83.1)
18.5–24.9	58.3 (51.1, 69.9)	63.9 (55.7, 73.2)	76.2 (62.2, 94.6)	47.1 (34.0, 64.6)	64.0 (48.7, 85.3)	63.6 (54.5, 80.9)
25.0–29.9	56.0 (43.4, 69.0)	65.1 (49.5, 75.3)	79.1 (65.0, 96.9)	49.5 (34.8, 72.0)	58.1 (44.0, 76.7)	71.8 (54.6, 77.2)
≥30.0	49.7 (36.9, 62.4)	59.1 (43.5, 75.4)	72.8 (57.4, 93.2)	44.5 (29.0, 66.0)	55.3 (40.8, 76.4)	60.7 (43.6, 78.9)

^1^ Mobile examination center (MEC) subgroup weights used for analysis. ^2^ Measured for male and female participants aged ≥2 years. BMI, body mass index; 25(OH)D, 25-hydroxyvitamin D.

**Table 4:** Weighted median (25th and 75th percentiles) 25(OH)D for the U.S. population, stratified by BMI: NHANES 2017–2018 ^1^.

		25(OH)D (nmol/L)			
		Weighted Median (25th and 75th Percentiles)			
		BMI (kg/m^2^) ^2^			
	N ^3^	<18.5	18.5–24.9	25.0–29.9	≥30.0
Age (years) ^4^					
20–39	1476	61.3 (41.4, 70.4)	63.7 (48.6, 78.4)	63.2 (48.7, 79.2)	57.2 (42.8, 71.3)
40–59	1557	70.8 (44.5, 97.5)	77.2 (59.5, 98.0)	72.7 (58.9, 88.1)	66.7 (50.5, 82.9)
≥60	1873	59.0 (40.3, 80.5)	92.7 (69.2, 112.0)	90.6 (68.9, 110.0)	84.4 (60.0, 106.0)
Gender					
Male	3489	70.9 (59.9, 83.3)	65.5 (51.5, 81.3)	70.6 (55.5, 86.0)	64.0 (49.1, 82.8)
Female	3693	69.6 (55.1, 84.1)	72.8 (56.8, 93.1)	76.7 (55.6, 101.0)	65.6 (47.2, 89.6)

^1^ Mobile examination center (MEC) subgroup weights used for analysis. ^2^ Measured for male and female participants aged ≥2 years. ^3^ Represents observed participants and not a weighted N. ^4^ Categories for age <20 years were not stratified by BMI due to the limited number of observations in these groups and were excluded from this analysis. BMI, body mass index; 25(OH)D, 25-hydroxyvitamin D.

**Table 5:** Weighted median (25th and 75th percentiles) 25(OH)D for the U.S. population, stratified by age: NHANES 2017–2018 ^1^.

25(OH)D (nmol/L)						
Weighted Median (25th and 75th Percentiles)						
Age (years)						
	0–4	5–11	12–19	20–39	40–59	≥60
Gender						
Male	77.6 (65.3, 87.1)	68.2 (59.1, 82.4)	64.1 (51.4, 79.0)	58.4 (45.5, 71.2)	69.6 (54.5, 83.8)	79.0 (60.4, 102.0)
Female	75.4 (63.6, 88.4)	66.7 (53.7, 78.0)	62.2 (46.8, 79.6)	61.7 (46.7, 78.1)	71.5 (53.8, 91.9)	92.2 (69.1, 110.0)

^1^ Mobile examination center (MEC) subgroup weights used for analysis. 25(OH)D, 25-hydroxyvitamin D.

**Table 6:** Weighted median (25th and 75th percentiles) 25(OH)D for the U.S. population, stratified by age, gender, and race/ethnicity: NHANES 2017–2018 ^1^.

	25(OH)D (nmol/L)						
	Weighted Median (25th and 75th Percentiles)						
Race/ Ethnicity	Overall	Mexican American	Hispanic non-Mexican	Non-Hispanic White	Non-Hispanic Black	Non-Hispanic Asian	Another Race
**Male**							
Age (years)							
0–4	77.6 (65.3, 87.1)	61.1 (55.0, 78.4)	73.7 (63.1, 77.4)	81.2 (70.6, 88.7)	64.7 (47.3, 82.1)	82.0 (65.3, 87.2)	74.3 (68.9, 85.5)
5–11	68.2 (59.1, 82.4)	64.4 (57.3, 71.1)	65.5 (56.1, 69.6)	76.4 (66.1, 90.2)	58.7 (45.4, 71.1)	64.9 (56.5, 70.6)	67.1 (57.3, 80.7)
12–19	61.2 (48.8, 73.6)	55.4 (41.8, 64.1)	57.1 (47.9, 67.0)	69.0 (60.1, 80.4)	45.7 (36.1, 54.0)	46.2 (38.8, 60.1)	60.4 (47.9, 74.0)
20–39	58.4 (45.5, 71.2)	50.7 (38.1, 59.9)	56.8 (46.2, 70.6)	64.3 (51.5, 76.1)	40.2 (28.3, 55.1)	49.7 (34.7, 58.4)	59.7 (44.5, 73.0)
40–59	69.6 (54.5, 83.8)	57.7 (42.7, 68.9)	66.7 (39.7, 78.1)	75.8 (64.5, 88.5)	45.8 (33.1, 62.9)	61.0 (47.3, 76.6)	64.5 (51.5, 77.2)
≥60	79.0 (60.4, 102.0)	65.2 (48.4, 84.9)	67.2 (51.8, 82.8)	85.7 (63.5, 103.0)	61.3 (41.2, 89.3)	80.7 (66.7, 96.2)	73.5 (59.7, 110.0)
**Female**							
0–4	75.4 (63.6, 88.4)	63.6 (59.2, 77.8)	71.4 (64.9, 82.0)	82.0 (72.4, 93.4)	57.2 (48.8, 73.0)	56.3 (49.1, 70.1)	77.4 (60.8, 108.0)
5–11	66.7 (53.7, 78.0)	59.1 (50.4, 68.1)	62.4 (53.9, 73.8)	75.2 (68.5, 86.8)	49.5 (39.3, 61.2)	56.3 (48.4, 62.2)	67.9 (57.7, 77.7)
12–19	60.0 (45.9, 73.0)	49.6 (37.8, 59.8)	55.9 (39.7, 59.2)	70.7 (59.1, 84.1)	38.5 (26.9, 48.7)	52.7 (40.4, 62.6)	63.6 (47.3, 68.3)
20–39	61.7 (46.7, 78.1)	49.3 (39.3, 64.3)	57.8 (49.1, 69.4)	71.9 (57.7, 87.7)	38.9 (27.1, 54.7)	50.9 (40.5, 64.5)	60.7 (42.6, 79.2)
40–59	71.5 (53.8, 91.9)	54.8 (44.7, 64.3)	69.4 (55.1, 78.8)	77.0 (58.6, 98.0)	55.5 (30.7, 75.6)	71.9 (55.9, 93.5)	72.4 (59.3, 89.7)
≥60	92.2 (69.1, 110.0)	74.3 (57.0, 90.1)	75.4 (62.1, 99.7)	93.9 (73.0, 112.0)	79.1 (47.3, 103.0)	88.2 (69.9, 112.0)	71.8 (61.1, 106.0)

^1^ Mobile examination center (MEC) subgroup weights used for analysis. 25(OH)D, 25-hydroxyvitamin D.

**Table 7:** Results from a mutually adjusted multiple linear regression model of total 25(OH)D, stratified by race/ethnicity: NHANES 2017–2018 ^1^.

	Fully Adjusted Model ^2^ N = 3772	Mexican American ^3^ N = 498	Hispanic Non-Mexican ^3^ N = 341	Non- Hispanic White ^3^ N = 1467	Non- Hispanic Black ^3^ N = 872	Non- Hispanic Asian ^3^ N = 388	Another Race ^3^ N = 206
	β (95% CI) (nmol/L)						
Age (years)							
20–39	0.0 (Ref)	0.0 (Ref)	0.0 (Ref)	0.0 (Ref)	0.0 (Ref)	0.0 (Ref)	0.0 (Ref)
40–59	5.9 (2.6, 9.1)	2.9 (−2.4, 8.1)	3.5 (−5.0, 12.0)	5.4 (0.6, 10.2)	8.1 (3.6, 12.6)	11.2 (3.6, 18.8)	8.7 (−2.2, 19.6)
60 and over	13.0 (9.7, 16.2)	15.9 (11.8, 20.0)	9.1 (3.0, 15.2)	11.9 (7.5, 16.3)	22.1 (18.3, 25.9)	19.0 (9.9, 28.2)	15.3 (6.7, 24.0)
Gender							
Male	0.0 (Ref)	0.0 (Ref)	0.0 (Ref)	0.0 (Ref)	0.0 (Ref)	0.0 (Ref)	0.0 (Ref)
Female	2.6 (−0.4, 5.6)	−0.4 (−5.8, 4.9)	1.3 (−3.8, 6.5)	2.8 (−1.4, 6.9)	1.6 (−4.1, 7.2)	6.4 (3.0, 9.7)	−0.2 (−7.4, 6.9)
BMI (kg/m^2^) ^4^							
<18.5	−0.8 (−10.0, 8.3)	NA	−23.1 (−31.9, −14.3)	−0.8 (−13.1, 11.5)	−3.8 (−18.7, 11.1)	8.8 (−5.9, 23.6)	39.1 (−11.7, 89.9)
18.5–24.9	0.0 (Ref)	0.0 (Ref)	0.0 (Ref)	0.0 (Ref)	0.0 (Ref)	0.0 (Ref)	0.0 (Ref)
25.0–29.9	−1.2 (−3.5, 1.1)	−3.0 (−10.4, 4.4)	−2.3 (−8.8, 4.3)	−0.8 (−3.7, 2.1)	4.0 (−1.3, 9.3)	−3.4 (−9.5, 2.7)	−6.9 (−21.8, 8.0)
≥30.0	−5.4 (−8.6, −2.1)	−8.4 (−16.1, −0.6)	−6.7 (−12.3, −1.0)	−4.1 (−8.4, 0.2)	−3.5 (−6.5, −0.5)	−6.4 (−14.2, 1.4)	−10.7 (−23.2, 1.8)
Educational Attainment							
<High school	−1.3 (−6.5, 4.0)	−1.6 (−5.8, 2.6)	−2.5 (−10.7, 5.7)	0.2 (−7.1, 7.4)	−7.3 (−12.6, −2.0)	−3.3 (−12.5, 5.8)	1.8 (−13.2, 16.8)
High school grad/GED or some college/AA degree	0.0 (Ref)	0.0 (Ref)	0.0 (Ref)	0.0 (Ref)	0.0 (Ref)	0.0 (Ref)	0.0 (Ref)
≥College graduate	2.2 (−1.8, 6.3)	6.6 (−1.0, 14.2)	1.8 (−5.0, 8.5)	2.7 (−3.2, 8.6)	−0.3 (−4.4, 3.7)	4.4 (0.4, 8.4)	−7.7 (−16.0, 0.7)
Supplement use mcg (IU/day)							
<15 mcg (600 IU)	0.0 (Ref)	0.0 (Ref)	0.0 (Ref)	0.0 (Ref)	0.0 (Ref)	0.0 (Ref)	0.0 (Ref)
16–25 (601–1000)	11.8 (7.5, 16.1)	21.6 (10.5, 32.8)	20.7 (9.7, 31.7)	8.6 (3.8, 13.4)	17.3 (13.0, 21.5)	17.3 (13.1, 21.6)	23.1 (11.9, 34.3)
≥26 (>1000)	30.0 (26.7, 33.3)	29.4 (21.9, 36.8)	35.1 (30.2, 40.0)	28.8 (24.8, 32.7)	33.8 (26.1, 41.5)	38.3 (28.9, 47.6)	21.3 (8.6, 34.0)
Dietary intake mcg (IU/day)							
<15 (600)	0.0 (Ref)	0.0 (Ref)	0.0 (Ref)	0.0 (Ref)	0.0 (Ref)	0.0 (Ref)	0.0 (Ref)
16–25 (601–1000)	7.7 (3.9, 11.4)	4.2 (−2.0, 10.4)	−5.6 (−16.4, 5.2)	9.3 (5.3, 13.3)	12.6 (1.7, 23.5)	10.2 (0.1, 20.2)	−10.6 (−19.8, −1.4)
≥26 (>1000)	4.1 (−3.0, 11.2)	17.2 (−3.4, 37.7)	3.6 (−6.8, 14.1)	−1.5 (−13.0, 10.0)	9.4 (−4.5, 23.3)	8.7 (−12.2, 29.6)	16.1 (0.7, 31.5)
Season							
1 November–30 April (Winter)	0.0 (Ref)	0.0 (Ref)	0.0 (Ref)	0.0 (Ref)	0.0 (Ref)	0.0 (Ref)	0.0 (Ref)
1 May–31 October (Summer)	8.9 (5.7, 12.1)	4.2 (−0.8, 9.3)	6.7 (2.4, 11.0)	10.5 (6.5, 14.4)	5.6 (0.3, 10.9)	2.5 (−0.9, 6.0)	9.0 (−1.4, 19.5)
Smoking Status ^5^							
Current	−3.7 (−7.0, −0.4)	−2.6 (−10.3, 5.1)	−11.3 (−15.8, −6.8)	−2.6 (−6.4, 1.1)	−7.2 (−11.9, −2.5)	−1.0 (−9.1, 7.1)	−5.9 (−18.1, 6.3)
Former	0.8 (−2.9, 4.6)	3.6 (−0.6, 7.8)	5.7 (−1.5, 13.0)	0.7 (−4.8, 6.2)	0.0 (−6.7, 6.7)	−3.0 (−10.1, 4.1)	−2.3 (−7.6, 3.0)
Never	0.0 (Ref)	0.0 (Ref)	0.0 (Ref)	0.0 (Ref)	0.0 (Ref)	0.0 (Ref)	0.0 (Ref)
Alcohol Use ^5^							
Yes	−1.2 (−4.5, 2.1)	1.2 (−5.0, 7.4)	1.1 (−6.3, 8.5)	−1.2 (−6.2, 3.9)	−4.4 (−9.0, 0.1)	−4.5 (−9.7, 0.6)	2.0 (−6.2, 10.1)
No	0.0 (Ref)	0.0 (Ref)	0.0 (Ref)	0.0 (Ref)	0.0 (Ref)	0.0 (Ref)	0.0 (Ref)
Diabetes							
Yes	−1.3 (−5.4, 2.9)	0.7 (−6.0, 7.5)	−3.0 (−16.3, 10.3)	−3.1 (−8.8, 2.5)	1.4 (−5.1, 7.8)	0.6 (−5.6, 6.8)	2.8 (−8.4, 14.1)
No	0.0 (Ref)	0.0 (Ref)	0.0 (Ref)	0.0 (Ref)	0.0 (Ref)	0.0 (Ref)	0.0 (Ref)
Kidney Disease ^6^							
Yes	6.5 (1.2, 11.7)	4.4 (−13.3, 22.1)	1.7 (−8.6, 12.1)	4.0 (−2.8, 10.8)	11.2 (2.4, 20.1)	10.6 (−9.1, 30.4)	30.2 (11.4, 48.9)
No	0.0 (Ref)	0.0 (Ref)	0.0 (Ref)	0.0 (Ref)	0.0 (Ref)	0.0 (Ref)	0.0 (Ref)

^1^ Diet and supplement subgroup weights used. ^2^ β (95% CI) for race/ethnicity: Mexican American: −13.0 (−17.7, −8.3); Hispanic non-Mexican: −10.0 (−13.1, −6.8); non-Hispanic White: 0.0 (ref); non-Hispanic Black: −19.4 (−21.7, −17.2); non-Hispanic Asian: −12.1 (−15.5, −8.7); Another Race: −7.7 (−13.1, −2.4). ^3^ *p*-value for the interaction term between race/ethnicity and all predictive factors: race×age: <0.001; race×gender: 0.11; race×BMI: <0.001; race×education: 0.011; race×supplement use: <0.001; race×dietary intake: <0.001; race×season: 0.23. ^4^ This variable was measured for male and female participants ≥2 years. ^5^ Measured for male and female participants aged ≥18 years. ^6^ Measured for male and female participants aged ≥20 years. BMI, body mass index; IU, International Unit; 25(OH)D, 25-hydroxyvitamin D.

## Data Availability

Data described in the manuscript are publicly available, and the analytic code will be made available upon reasonable request to the corresponding author.

## Supplementary Materials

The following supporting information can be downloaded at: https://www.mdpi.com/article/10.3390/nu16193414/s1, Figure S1: 25(OH)D, stratified by age, gender, and race/ethnicity in NHANES 2017–2018. The blue and red dots represent the weighted median 25(OH)D levels, and the lines represent the corresponding 25th and 75th percentiles; Table S1: Weighted median (25th and 75th percentiles) 25(OH)D for the U.S population, stratified by age: NHANES 2017–2018.
