# Supplementary material for: Vitamin D Levels in the United States: Temporal Trends (2011–2018) and Contemporary Associations with Sociodemographic Characteristics (2017–2018)
Source: Nutrients. 2024 Oct 9;16(19):3414. doi: 10.3390/nu16193414 (PMC11478591; doi:10.3390/nu16193414)
Supplement: Supplementary file 1 [file nutrients-16-03414-s001.zip › nutrients-3220196-supplementary.pdf]

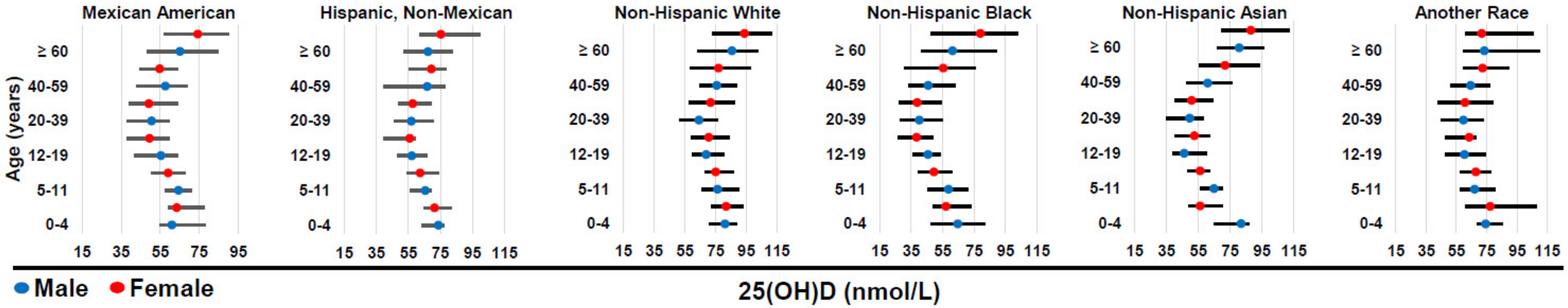

Figure S1. 25(OH)D, stratified by age, gender, and race/ethnicity in NHANES 2017–2018. The blue and red dots represent the weighted median 25(OH)D levels, and the lines represent the corresponding 25th and 75th percentiles.

Table S1. Weighted median (25th and 75th percentiles) 25(OH)D for the U.S population, stratified by age: NHANES 2017–2018.[1]

		25(OH)D (nmol/L)						
		Weighted Median (25th and 75th percentiles)						
			Age (years)					
	N[2]	Overall	0-4	5-11	12-19	20-39	40-59	≥60
**Dietary vitamin D mcg (IU)** N=6,617[2]								
<15 (600)	6,347	68.9 (52.1, 87.4)	76.0 (64.0, 87.1)	69.0 (56.8, 82.8)	60.5 (46.8, 73.6)	59.7 (44.8, 74.8)	70.9 (54.1, 87.8)	86.8 (64.6, 107.0)
16-25 (601-1,000)	199	69.3 (58.1, 85.3)	79.5 (73.6, 109.0)	71.6 (63.5, 87.2)	67.8 (45.7, 72.0)	65.6 (58.2, 72.2)	69.3 (55.7, 85.3)	94.7 (80.4, 107.0)
26+ (>1,000)	71	75.1 (53.1, 95.5)	NA[3]	NA[3]	83.8 (52.1, 92.2)	74.3 (48.7, 85.6)	81.3 (47.0, 98.4)	79.8 (53.8, 107.0)
**Vitamin D supplements mcg (IU)** N=6,625[2]								
<15 (600)	5,248	63.6 (48.4, 78.1)	75.7 (63.1, 86.4)	69.0 (56.4, 82.3)	59.7 (45.9, 71.8)	58.3 (44.2, 71.9)	65.4 (47.9, 79.8)	69.5 (55.0, 87.3)
16-25 (601-1,000)	681	80.1 (66.4, 96.4)	85.0 (72.6, 107.0)	72.2 (61.9, 90.1)	71.6 (60.4, 84.9)	70.8 (56.5, 83.7)	76.8 (64.5, 90.3)	91.3 (78.3, 106.0)
26+ (>1,000)	696	100.0 (82.8, 120.0)	NA[3]	NA[3]	77.7 (65.7, 114.0)	93.7 (69.8, 111.0)	91.9 (75.2, 112.0)	107.0 (92.2, 126.0)

[1]Diet and supplement subgroup weights used for analysis.

[2]Represents observed participants and not a weighted N.

[3]Not applicable due to only 1 or fewer observations within this group.
